# Conscious sedation teaching in dental schools of the United Kingdom and Ireland: an update

**DOI:** 10.1038/s41415-025-8400-5

**Published:** 2025-07-11

**Authors:** Kathryn Taylor, Anna Dargue, Anna Vincent

**Affiliations:** 41415379437001 University of Central Lancashire Dental School, Preston, UK; Member, Dental Sedation Teachers Group, UK; 41415379437002 Bristol Dental School, University of Bristol, 1 Trinity Quay, Avon Street, Bristol, BS2 0PT, UK; 41415379437003 University of Central Lancashire Dental School, Preston, UK

## Abstract

**Aim** To examine the current state of conscious sedation teaching to undergraduates in the dental schools of the United Kingdom (UK) and Ireland.

**Background** In 2000, Leitch and Girdler published ‘A survey of the teaching of conscious sedation in the United Kingdom and Ireland' in the *British Dental Journal*, which gave insight into the undergraduate experience and the teaching of conscious sedation in UK and Ireland at that time. In October 2022, the Dental Sedation Teachers Group (DSTG) adapted this survey for the wider dental team to evaluate current undergraduate conscious sedation education in dental schools in the UK and Ireland.

**Methods** A survey was adapted and piloted by two DSTG school representatives. This was distributed via email to the DSTG school representatives of UK and Irish dental schools, with repeat emails sent to non-responders.

**Results** In total, 13 out of 16 schools responded. Most sedation teaching was led by staff from oral surgery and paediatric dentistry (nine schools). This survey included the wider dental team; sedation training was delivered by staff from hygiene and therapy departments in three schools.

**Conclusion** Decreased sedation experience was observed in most schools over the last 25 years. Currently, there is limited experience of undergraduate sedation in most schools.

## Key points

- Gives insight into the provision of conscious sedation teaching at an undergraduate level.
- Increases awareness of the reduction in the level of conscious sedation teaching at an undergraduate level.
- Examines the impact of a changing educational environment on undergraduate conscious sedation teaching.

## Background

In 1998, for the first time, Leitch and Girdler undertook a national survey on the undergraduate teaching and clinical experience of conscious sedation among dental students in the United Kingdom (UK) and Ireland.^1^ This survey was sent to staff involved in the teaching of undergraduate conscious sedation at each dental school, with an 81% response rate; some schools also provided a response from dental students (44% response). The results of the survey demonstrated variation in sedation teaching with little practical experience, particularly in intravenous (IV) sedation.^1^ A follow-up national survey was undertaken three years later, which showed an improvement in undergraduate sedation teaching, with an increase in didactic teaching, as well as both observation and hands-on experience of intravenous sedation.^2^

Undergraduate dental students are not required to be competent in the delivery of conscious sedation upon graduating: this requires postgraduate training. The General Dental Council (GDC) states, in the 2015 Preparing for Practice document, that by qualification, dentists should be able to ‘prevent, diagnose and manage patient anxiety appropriately, effectively and safely' as well as ‘evaluate the risks and benefits of treatment under conscious sedation and make appropriate referrals'.^3^ This was reinforced by the Scottish Dental Clinical Effectiveness Programme who stated in 2017 that ‘dentists should be able to assess the patient and refer for conscious sedation if appropriate'.^4^ Correspondingly, the Association for Dental Education in Europe (ADEE) lists, in the ‘The graduating European dentist' learning outcomes, that they should be able to apply knowledge of ‘the role of and indications for the use of sedation, particularly in the management of anxious and uncooperative patients, including those with systemic disease'.^5^ To achieve this, undergraduates need to have knowledge and clinical experience of a range of conscious sedation techniques that can be used for anxiety management.

Similar to dental students, student hygienists and therapists require additional postgraduate training to undertake inhalation sedation in practice. There are also similar learning outcomes required by the GDC in terms of assessing and managing patient anxiety, as well as being able to ‘explain the risks and benefits of treatment under…conscious sedation'.^3^

It is now nearly a quarter of a century since the first national survey and members of the dental team have changed. Therefore, the Dental Sedation Teachers Group (DSTG) adapted the previous surveys to include the wider dental team.

The aim of this survey was to ascertain the current state of undergraduate education and training in conscious sedation in UK and Irish dental schools.

## Materials and methods

The study was designed as a prospective, questionnaire-based survey. The questionnaire requested information on the quantity and type of didactic and clinical teaching, as well as asking the staff member to assess the overall quality of their sedation teaching, and as such, ethics approval was not necessary. Dental students were not included as previous response rates were poor and DSTG did not look to assess the students' perceptions of their teaching. The questionnaire was designed to ensure brevity and ease of completion by teaching staff, which was initially piloted by two DSTG school representatives and amended. A member of staff was selected from all 14 UK and two Irish dental schools, which were the schools' nominated representative of the DSTG. The survey was emailed via Google Forms (Table 1) in October 2022. Repeat emails were sent (at six weeks and three months) to increase the response rate and results were collated. All respondents consented to participate.

**Table 1:** Questionnaire to staff involved in teaching undergraduate sedation in UK and Ireland

1.	Which dental school do you represent?	
2.	Which departments contribute to sedation teaching in your institution?	Oral surgery Paediatric dentistry Sedation Anaesthesia Restorative Hygiene and therapy Other
3.	In which undergraduate course is sedation taught?	Dentistry Hygiene and therapy Therapy
4.	When is sedation taught to your undergraduates? i.e., theory in Year 4 BDS, practical sessions in Year 5	
5.	How many students do you have in each year for dentistry, hygiene and therapy, and therapy?	
6.	In what form are the theoretical aspects of sedation taught?	Seminars Lectures Seminars and lectures Other modalities
7.	Please expand on your response to the previous question? How many hours of sedation lectures/seminars/workshops do you arrange for the students? What other teaching modalities do you use?	
8.	What experience would you expect your students to get in IV sedation i.e., number of observed cases, number of cases where students deliver sedation to a patient?	
9.	What experience would you expect your students to get in inhalation sedation i.e., number of observed cases, number of cases where students deliver sedation?	
10.	How would you rate the adequacy of intravenous sedation teaching in your institution?	1-5 (not very good - excellent)
11.	How would you rate the adequacy of inhalation teaching in your institution?	1-5 (not very good - excellent)
12.	Is there anything else that you would like to add? All responses will be anonymised	

Data from the returned questionnaires were entered into an Excel spreadsheet and simple data calculations performed.

## Results

In total, 13 out of 16 dental schools (identified as schools A to M) in the UK and Ireland completed the survey (81% response rate). Most sedation teaching was performed by a combination of staff from the departments of oral surgery and paediatric dentistry (nine schools). Two schools were taught by oral surgery staff alone and there were four dental schools with specific sedation departments which delivered undergraduate sedation education in conjunction with other departments. This survey included the wider dental team and sedation training was delivered by staff in hygiene and therapy departments in three of the schools that responded. None of the schools who responded in this survey had anaesthetic staff involvement in sedation teaching.

**Fig. 1:**
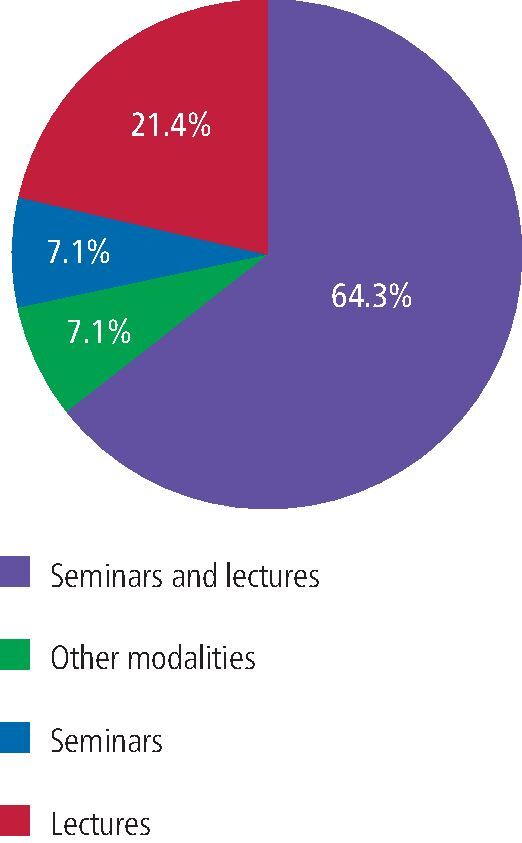
Didactic teaching of sedation in UK and Ireland dental schools

Most didactic teaching (64%) was provided by a combination of lectures and seminars (Fig. 1). There was evidence of enhanced experience in some dental schools, with supplementary recordings of patient treatment sessions; interactive sessions; student-led case-based discussions; and workshops, including cannulation, monitoring and use of inhalation sedation machines. Most schools reported 5-6 hours of teaching supplemented with practical skills teaching. This was primarily delivered in Year 4 of the undergraduate dental degree programme (extending to Year 5 for practical clinical experience) or Year 2 of the hygiene and therapy programmes. School F delivered all didactic and clinical teaching in Year 5 and School J demonstrated experiential learning from Year 2-5 of their integrated clinical programme.

There was great variability recorded in this survey of the student experience of both intravenous and inhalation sedation across different dental schools (Table 2). One school (H) provided excellent hands-on experience for their students in intravenous sedation. Several schools were unsure of their students' level of involvement in sedation. Since the published results of the previous surveys, there has been a notable decrease in all types of inhalation and intravenous sedation experience provided by most schools (Table 3). A student's experience of providing inhalation and intravenous sedation is seen to be particularly low, with an average of 0.8 inhalation cases and 0.3 intravenous cases.

**Table 2:** Number of intravenous/inhalation cases experienced by each student at each dental school in UK and Ireland

**Dental school**	**Number of intravenous sedation cases per student**	**Number of inhalation sedation cases per student**
A	Not sure	Not sure
B	Four observed cases	Very little
C	Six observed cases with 1-2 sedation delivered	1-2 observed
D	No IV observed	Two inhalation cases for requirements
E	1-2 operator and assisting	3-4 operating and assisting
F	Maximum two observed	Maximum three cases observed only
G	Eight observed IV cases	Four observed cases
H	30-40 observed cases IV cannulation 2-3	None
I	Observation only in oral surgery and outreach	None
J	Three observed cases	2-3 cases each with treatment
K	None performed, one observed	No performed cases, average one observed
L	Not sure	Not sure
M	Variable numbers, observed only	Not sure. No specific requirements

**Table 3:** Comparison of findings from 1998,^1^ 2001^2^ and current 2022 surveys of sedation teaching in UK and Irish dental schools

	**Leitch and Girdler, 19981**		**Leitch and Jauhar, 20012**		**Current DSTG survey**	
Response rate	13/16 (81%)		9/15 (60%)		13/16 (81%)	
Dental student response rate	7/16 (44%)		11/15 (73%)		-	
	From 13 schools in 1998 survey		From eight schools in 2001 survey		From 13 schools in 2022 survey	
Source of teaching	2/13 sedation departments Primarily oral surgery and paediatrics 3/13 involvement of anaesthetic departments		Largely multi-disciplinary 1/8 sedation department only 3/8 involvement of anaesthetic department		4/13 had sedation departments 9/13 oral surgery and paediatrics 2/13 oral surgery alone 3/13 teaching by hygiene and therapy staff	
Student year of sedation teaching	-		-		Primarily Year 4 and 5 clinical Year 2 hygiene and therapy	
	From seven schools with staff and student responses		From eight schools with staff and student responses		From 13 schools - staff responses only	
Type of didactic teaching per school	Mean 4.2 lectures (0-10) Mean 1.8 seminars (0-6) 5/7 didactic teaching		Mean 7.25 lectures (3-11) Mean 1.5 seminars (0-5) All had didactic teaching 4/8 seminars		64.3% seminars and lectures 21.4% lectures 7.1% seminars 7.1% other: recordings, interactive sessions, student-led CPD, workshops Most schools 5-6 hours	
Type of clinical teaching	Inhalation observed	Mean 5.1 (1-10)	Inhalation observed	Student 7 (0-17) Staff 3 (0-8)	Inhalation observed	0-4
	Inhalation performed	Mean 2.6	Inhalation performed	Student 4 (0-8) Staff 4 (0-8)	Inhalation performed	0.8
	Intravenous observed	Mean 4.4 (1-8)	Intravenous observed	Student 9 (2-19) Staff 8 (0-40)	Intravenous observed	0-40
	Intravenous performed (two schools)	Mean 1.1	Intravenous performed (eight schools)	Student 5 (0-8) Staff 4 (0-7)	Intravenous performed (three schools)	0.3
Staff member rated overall quality	2/7: very satisfactory 1/7: satisfactory 4/7: unsatisfactory		4/6: satisfactory 2/6: unsatisfactory		Inhalation	Intravenous
					1/13 excellent 3/13 6/13 2/13 1/13 not very good	1/13 excellent 3/13 7/13 1/13 1/13 not very good
Student experience	1/7: very satisfactory 4/7: satisfactory 1/7: average 1/7: unsatisfactory		1/6: very satisfactory 4/6: satisfactory 1/6: average		-	

**Fig. 2:**
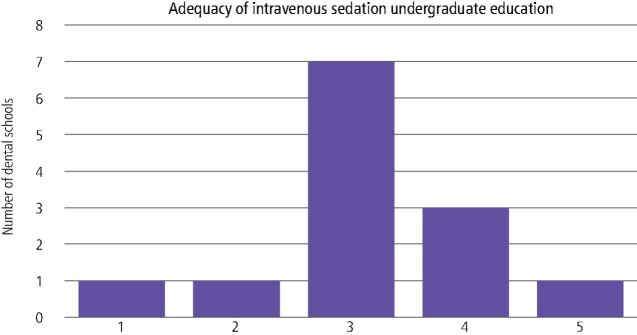
Self-reported adequacy of intravenous sedation teaching (range: 1-5 [not very good - excellent])

**Fig. 3:**
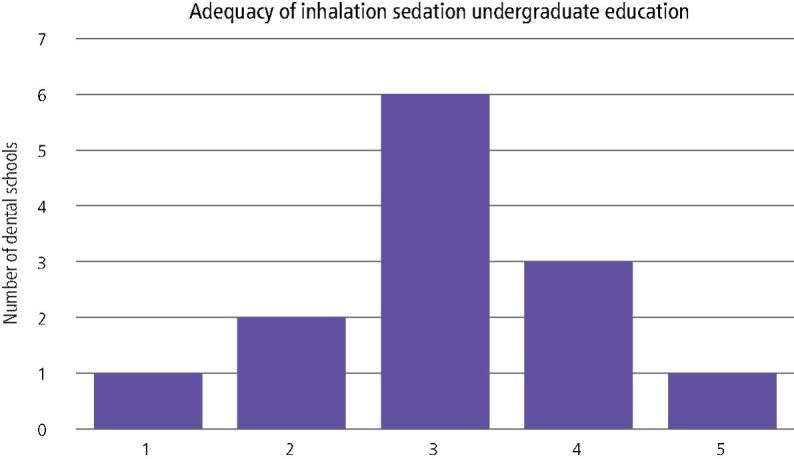
Self-reported adequacy of inhalation sedation teaching (range: 1-5 [not very good - excellent])

Respondents were asked on the adequacy of their intravenous (Fig. 2) and inhalation sedation teaching (Fig. 3). Most sedation teachers believed their schools inhalation and intravenous sedation teaching was adequate but could be improved.

## Discussion

There was an 81% response rate from staff at dental schools in the UK and Ireland, which was identical to the 1998 survey and an improvement on the 2001 survey, which only had a 60% response rate. As was demonstrated in the 1998 and 2001 surveys, there was a wide variation in the undergraduate conscious sedation education experience.^1^^,^^2^ Table 3 compares the three surveys regarding sedation teaching and although there are subtle differences in the questionnaires, there is little evidence of improvement. It is evident from our recent survey that students have reduced didactic and practical teaching of conscious sedation and this has worsened since 1998. The dental team has also expanded with direct access, so dental care professionals also require teaching and experience of anxiety management techniques. The background of dental sedation teachers appears to reflect this change, with three dental schools now having staff involvement from hygiene and therapy departments. Interestingly, three dental schools were noted to no longer have the involvement of anaesthetic colleagues. The 1997 GDC dental curriculum, while now out of date, specifically recommended that the collaboration between dental teachers and anaesthetic departments was a positive one, so this change appears disappointing.^1^ Compared to 1998, two further schools have dedicated sedation departments (n = 4). This is promising but raises the question as to why the experience of students has reduced. Several respondents believed that the COVID-19 pandemic had had a deleterious effect on student experience and that dental schools now place less importance on the management of dental anxiety. Most respondents believed that the undergraduate experience of conscious sedation education would not recover to pre-pandemic levels. Results demonstrated that respondents felt that undergraduate training was adequate but they would like to offer an increased experience if resources could be identified for this.

### Educational dilemma

UK dental schools must follow the GDC learning outcomes in the Preparing for Practice document to suitably prepare undergraduate students for graduation and the transition to dental practice.^3^ However, these learning outcomes are not prescriptive and so allow universities flexibility in fulfilling them. This has the side effect of variable national interpretation across dental schools, especially in relation to the need for practical experience.^6^ Similar issues are reported in Spanish dental schools; the ambiguity in the educational requirements causes a dilemma in the amount and content of sedation training a dental student requires.^7^ Authors in the UK and United States (USA) have underlined the low curricular priority for sedation teaching in dental schools.^1^^,^^8^ Other barriers to increased undergraduate sedation experience have been identified as increasing student numbers with larger-sized groups, lower staffing levels, high patient non-attendance rates, student limitations (e.g., needle phobia) and even bad weather.^9^^,^^10^ Furthermore, research has shown significant discrepancies between the hands-on experiences between clinical groups.^9^ It is our opinion, and that of other UK sedation teachers, that dental education providers should in fact be inspiring our future generation of dental graduates to undertake formal postgraduate training by offering them greater experiences as undergraduates.^6^ Studies in the UK and USA showed that students who had a greater clinical experience of sedation felt better prepared and more confident, as well as being more satisfied with the quality of teaching.^6^^,^^8^

### Need for sedation

It is widely recognised that NHS (National Health Service) primary dental care is facing a crisis, with patients having difficulty accessing treatment.^11^ For anxious patients, this can be exacerbated, where NHS resources are limited and conscious sedation is not offered in many primary care NHS practices.^12^ The most recent UK Adult Dental Health Survey (2011) showed 51% of the adult population experience low dental anxiety, 36% moderate levels and 12% high levels.^13^ UK dental graduates and the wider dental team need to be adequately prepared for managing the needs of anxious patients. Other authors across Europe and in the USA have also highlighted the persistence of fear and dental anxiety in the population and the need for sedation services within dentistry.^7^^,^^8^ In particular, it is vital that greater inhalation services are available to the child population as an alternative to general anaesthesia.^6^ In January 2023, the NHS produced the document Clinical standards for dental anxiety management, which describes the management of anxious dental patients in community and primary care settings.^14^ This guidance suggests that ‘GDC-registered members of the dental team with pre-registration training and experience should provide low-level anxiety management for all tiers, and patients with higher-level anxiety or requiring more invasive treatment should be referred to specialist providers'.^14^ Unfortunately, however, there are geographic differences in the support available in secondary care referral centres. The Getting It Right First Time (GIRFT) report in 2022 reviewed the sedation delivery pathway for hospital dentistry and found that only 49% of hospital trusts offer sedation services.^12^ The report also noted that where conscious sedation was not available, there was an increase in the use of general anaesthesia with associated costs and morbidity.^12^ There are also long patient waiting times for patients who have been referred to secondary care centres for treatment with conscious sedation. Some patients may select treatment in the private sector if they can afford it, but patients in lower socioeconomic groups are unable to afford this, promoting inequalities in access to dental care. It can be seen, therefore, that if more conscious sedation were available in primary care, this would be more cost-effective, have reduced patient morbidity and overall reduce the inequalities in the availability of dental care. Clearly, treating patients with conscious sedation techniques requires formal postgraduate training and clinical experience and this was clarified in 2015 (updated 2020) when the Intercollegiate Advisory Committee for Sedation in Dentistry guidelines were published.^15^ Graduates need to be able to assess and refer appropriately, and at least observe or assist with a range of modalities available for anxiety management. A study at Glasgow Dental School highlighted that with adequate resources and support, all students had valuable training in conscious sedation.^10^ In light of our data, however, it appears that most graduates will not get this opportunity; rather the emphasis is on encouraging postgraduates to complete accredited training to manage these patients in a safe and effective manner. Progress needs to be made in providing these opportunities for undergraduates and a continuation of this into foundation training schemes.

## Conclusion

It is disappointing that practical experience of conscious sedation for undergraduates in the UK and Ireland has worsened since the original Leitch and Girdler publication nearly 25 years ago.^1^ This reflects increasing pressures on dental schools and resources. The GIRFT publication for sedation pathways in hospital dentistry was a positive move to enhance provision for anxious patients requiring dental treatment in secondary care services and it is hoped that secondary care conscious sedation services continue to develop.^12^ Academic providers, such as universities, DSTG and the Society for the Advancement of Anaesthesia in Dentistry, continue to provide undergraduate and postgraduate resources and training opportunities, but conscious sedation in NHS primary care needs to be adequately funded so that the needs of anxious patients requiring dental treatment are met.

## Data Availability

All data are available in the UCLan data repository: .
